# Collection of cells for single-cell RNA sequencing using high-resolution fluorescence microscopy

**DOI:** 10.1016/j.xpro.2021.100718

**Published:** 2021-08-05

**Authors:** Hendrika A. Segeren, Kiki C. Andree, Lisa Oomens, Bart Westendorp

**Affiliations:** 1 Department of Biomolecular Health Sciences, Faculty of Veterinary Medicine, Utrecht University, Utrecht, the Netherlands; 2 VYCAP B.V., Enschede, the Netherlands

**Keywords:** Cell Biology, Single Cell, Genomics, Microscopy

## Abstract

FACS sorting followed by single-cell RNA-sequencing (SORT-Seq) is a popular procedure to select cells of interest for single-cell transcriptomics. However, FACS is not suitable for measurement of subcellular distribution of fluorescence or for small samples (<1,000 cells). The VYCAP puncher system overcomes these limitations. Here, we describe a workflow to capture, image, and collect fluorescent human retina pigment epithelium cells for SORT-Seq using this system. The workflow can be used for any cell type with a diameter of ∼5–50 μm.

For complete details on the use and execution of this protocol, please refer to Segeren et al. (2020).

## Highlights

• Capture up to 6,400 living fluorescent-labeled single cells on a chip using air pressure
• High-magnification fluorescence microscopy and selection of cells of interest
• Punch single cells of interest into microwells using an ultrafine needle
• Microwell plate processing for single-cell RNA-sequencing using automated platform

## Graphical abstract

**Figure:**
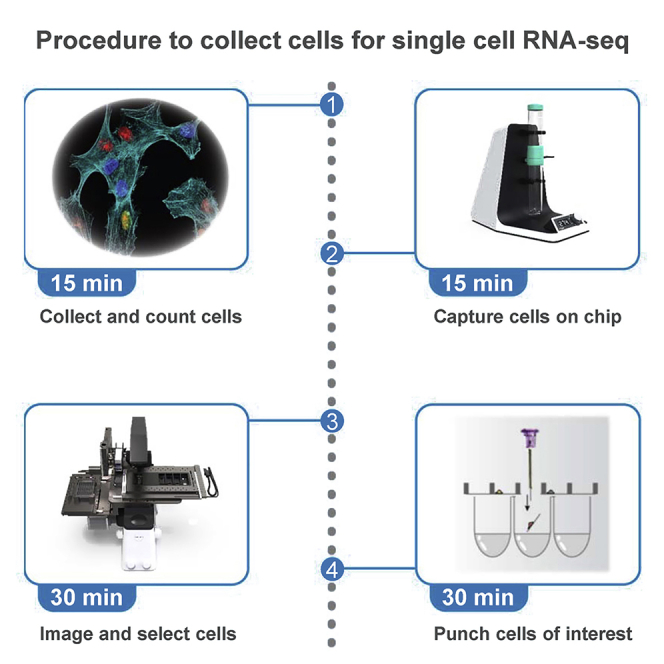

## Teaser

FACS sorting followed by single-cell RNA-sequencing (SORT-Seq) is a popular procedure to select cells of interest for single-cell transcriptomics. However, FACS is not suitable for measurement of subcellular distribution of fluorescence or for small samples (<1,000 cells). The VYCAP puncher system overcomes these limitations. Here we describe a workflow to capture, image, and collect fluorescent hTert-RPE1 cells for SORT-Seq using this system. The workflow can be used for any cell type with a diameter of ∼5–50 μm.

## Before you begin

**Figure 1:**
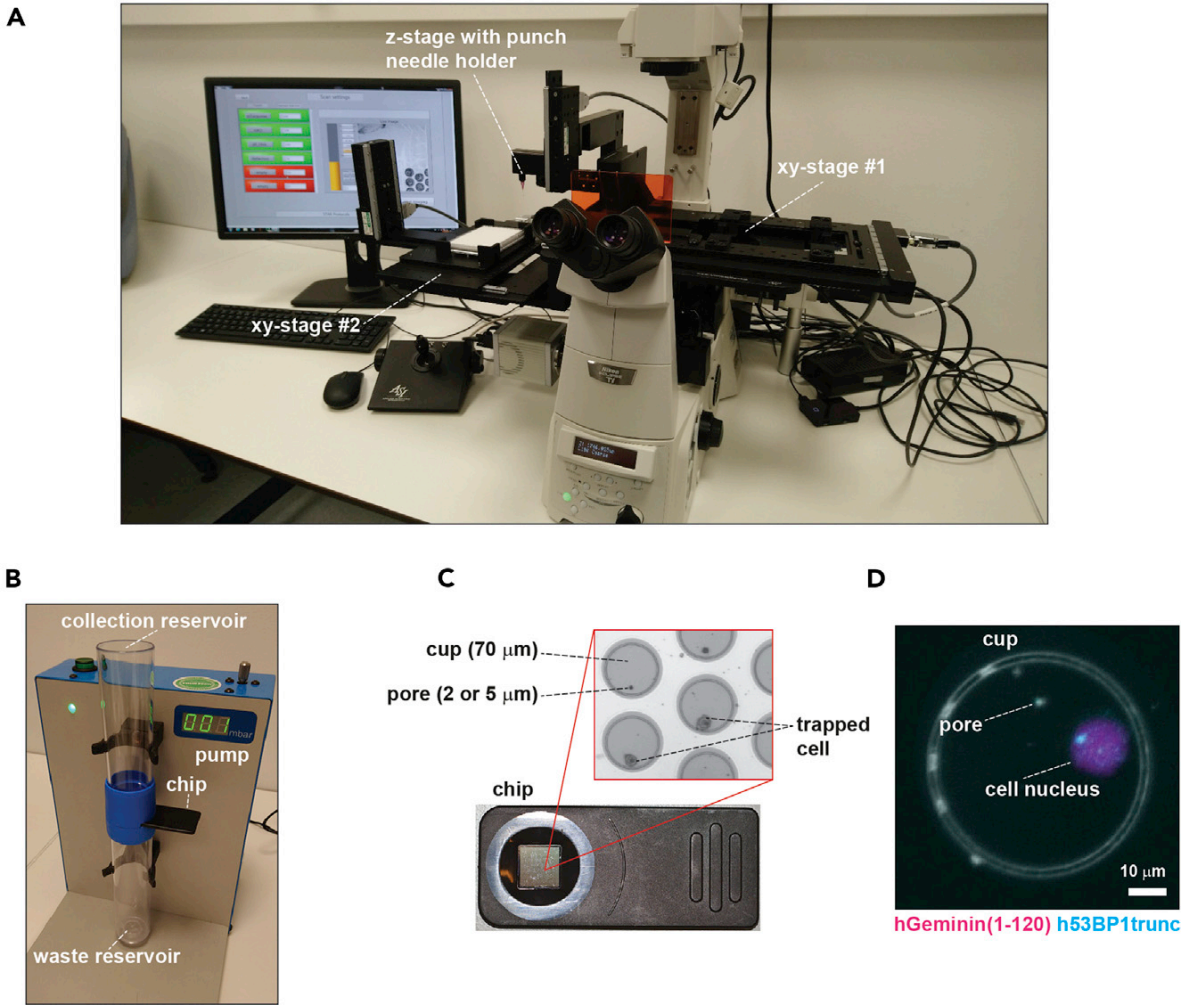
Collecting single cells for RNA-sequencing with the VYCAP needle puncher (A) Customized microscope system with dedicated software to isolate, select and punch single cells. The chip is placed on xy-stage #1, and the microwell plate is placed on xy-stage #2. (B) Pump system to apply a negative pressure on the chip to pull the single cell suspension from the collection reservoir towards the waste reservoir. (C) The cell capture chip. Inset: microscopic overview of the grid, which contains 6400 cups that each contain a pore. The chip acts as a sieve; single cells cannot pass through the pores. (D) Image showing cell with 53BP1 foci and nuclear expression of Geminin(1–120) captured on a VYCAP microwell chip. Images were taken with a 40× long working distance objective.

The platform used in this protocol has three main components, which are described in detail elsewhere (Stevens et al, 2018). Briefly, the first component is a customized microscope equipped with 2 xy stages, a z stage containing a needle for punching, and dedicated software (Figure 1A). The second component is a disposable microwell chip placed in a two-compartment reservoir (Figure 1B). The chip contains a grid with 80 × 80 microwells (referred to as cups hereafter) and act as a sieve in which cells are collected (Figure 1C). Each cup contains a pore with a diameter of either 2 or 5 μm. Fluid can pass through, but single cells are trapped on these pores. The third component is a vacuum pump to pull the cell suspension through the chip (Figure 1B).

The protocol below describes the specific steps for human retina pigment epithelium (hTERT RPE-1) cells carrying the FUCCI-system (Sakaue-Sawano et al, 2008). However, the protocol is suitable for or can be adapted to other adherent cell cultures or single cell suspensions. For example, we have also used this protocol to create single cell RNA-sequencing data from primary hepatocytes, as well as from colorectal carcinoma (HT29) and prostate cancer (PC-3 and LNCAP) cell lines.

Cell selection in the current protocol is done based on overall fluorescence intensity per nucleus. However, the system is equipped with a 40× long working distance objective and therefore well suited to analyze subcellular fluorescent signals, such as formation of fluorescent tagged truncated 53BP1 foci in response to replication stress-induced DNA damage (Figure 1D).

### Prepare reporter-carrying cells

**Timing: 2–4 weeks**

1. To create reporter-carrying cell lines, different cell lines and reporters can be used. We routinely use hTERT RPE-1 cells transduced with lentivirus carrying the cell cycle reporters mAG-hGeminin(1-110), mKO2-hCDT1(30-120), and mTurquoise2-53BP1trunc (Segeren et al, 2020).
  **CRITICAL:** At least one fluorescent marker must be present in all cells to enable automated selection of cells. Hoechst33342 or another DNA dye able to pass membranes of intact cells can be used for this purpose.
  ***Optional:*** Add a fluorescent lipophilic dye to allow simultaneous capture of two different conditions in one sequencing plate. For example drug versus control or knockout versus wild-type.
  a. Add 1 μL of the fluorescent dye DiIC18(5); 1,1′-dioctadecyl-3,3,3′,3′- tetramethylindodicarbocyanine, 4-chlorobenzenesulfonate salt (DiD) per mL medium to one of the two conditions.
  b. Gently swirl the culture dish, and place the cells back into the incubator for 1 h.

### Prepare the chip

**Timing: 1–3 h**

**Figure 2:**
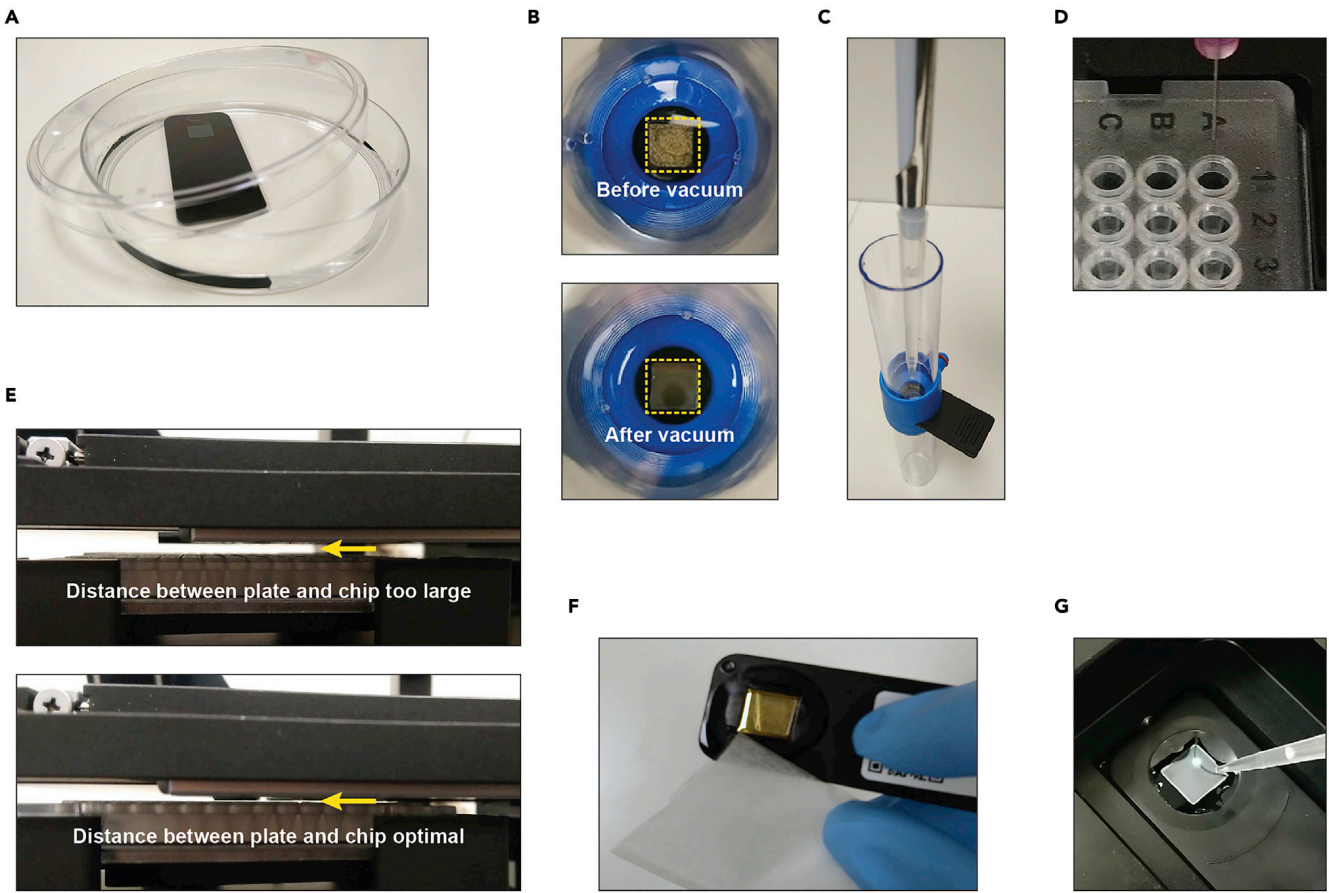
Preparing the cell capture chip (A) Immerse chip in 100% ethanol in a petri dish to prepare the air bubble removal step. (B) Chip viewed from above, before (upper image) and after (lower image) removal of air bubbles and re-mounting. The areas containing cups are marked by the yellow boxes. Note that the surface of the chip looks smooth and regular after bubble removal. After this step, make sure that the chip does not run dry again. (C) Re-mount the chip in the holder and gently pipet 1 mL of PBS on top. Avoid air bubble formation at all times. (D) Correct alignment of the microwell plate. The first well (A1) should be right underneath the tip of the needle. (E) The plateholder must be calibrated in the z-stage such that the distance between microwell plate and chip holder is not more than 1–2 mm (lower panel, yellow arrow). (F) Dry the backside of the chip by touching a corner with a piece of absorbing lens cleaning paper before mounting the chip on the microscope. Avoid touching the chip. (G) Pipet roughly 150–200 μL of PBS or serum-free medium on the chip as soon as it is mounted on the microscope.

***Note:*** Chips with different pore sizes are available. Choose the chip with appropriate pore size based on cell size and compatibility with downstream analysis. Default chips have a pore size of 5 μm and suffice for regular sized cells and evaluation of nuclear reporters.

2. Filter 100% analysis-grade ethanol and PBS using a 0.2 μm filter.
3. Remove the chip from the holder, and immerse it completely in a Petri dish containing filtered 100% analysis-grade ethanol (Figure 2A).

4. Place the Petri dish containing the chip immersed in ethanol in a vacuum chamber. Maintain the vacuum until the chip appears completely free from air bubbles (Figure 2B). This can take 0.5–3 h depending on the vacuum applied.
5. Place the chip back into the holder and gently pipet 1 mL of sterile, pre-filtered PBS on top of the chip (Figure 2C). Take care to produce no new bubbles.
6. To wash the chip with a 5 μm pore size, switch on the pump at 20 mbar negative pressure to pull the PBS through the pores of the chip. Increase the pressure to −100 mbar for chips with a pore size of 2 μm.
7. When the PBS is almost gone and only 100–200 μL of liquid still covers the chip, apply another 1 mL of PBS.
8. Repeat step 9.
9. Switch off pump when PBS is almost gone. Keep the chip covered with a small amount of PBS (∼200 μL).

**CRITICAL:** Avoid that the chip runs dry during steps 8–11. Otherwise new air bubbles will form, and block the pores of the microwell chip (Figure 2C). Don’t keep the pump running unattended and stop it as soon as the liquid surface is a few millimeters above the collection cups.

### Align the needle and the collection plate

**Timing: 10 min**

10. Start the Puncher and open the VYCAP Punch software.
11. Place the needle and wait at least 3 min to let it settle in its holder.
12. Select the desired objective and the type of collection device (384-well plate). Align the 384-well plate in xy-directions using the VYCAP software; the needle should be above the middle of well A1 (Figure 2D).
13. Align the 384-well plate in the z-direction using the VYCAP software; make sure that the distance between chip and plate is as short as possible (Figure 2E).
14. Calibrate the needle via the VYCAP software.
15. Adjust in the settings section of the VYCAP software the frequency of re-calibrations between the punches. The frequency of re-calibration is a trade-off between speed and accuracy, but we typically calibrate each 24 punches.

***Optional:*** Check proper alignment by punching a few wells of a clean, dry chip.

## Key resources table

**Table:** 

REAGENT or RESOURCE	SOURCE	IDENTIFIER
**Chemicals, peptides, and recombinant proteins**		

Vybrant DiD Cell-Labeling Solution	Thermo Fisher Scientific	Cat# V22887
Hoechst 33342	Thermo Fisher Scientific	Cat# H3570
Ethanol 100% analysis grade	Merck	Cat# 100983

**Deposited data**		

Single cell RNA-sequencing RPE-FUCCI cells	(Segeren et al, 2020)	GEO: GSE146759

**Experimental models: Cell lines**		

hTERT RPE-1-FUCCI	(Boekhout et al, 2016)	N/A
hTERT RPE-1-FUCCI-*E2F7/8*^*KO*^	(Yuan et al, 2019)	N/A

**Software and algorithms**		

FIJI (ImageJ)	http://fiji.sc	RRID:SCR_002285
Custom VYCAP image quantification macro	This paper	N/A

**Other**		

Puncher system	VYCAP	Cat# Ti2-PS
Pump unit	VYCAP	Cat# PU-250
Punch needles	VYCAP	Cat# PN-70
Disposable single cell microwell chips	VYCAP	Cat# FM75-510
CEL-Seq2 collection plates (384)	Single Cell Discoveries	N/A
Microseal ‘F’ PCR plate seal foil	Bio-Rad	Cat# MSF1001
Nalgene Syringe Filters 0.22 μm	Thermo Fisher Scientific	Cat# 723-2520

## Materials and equipment

For the cell capture, a VYCAP Puncher system is required. The system is equipped with dedicated software for imaging, cell selection and punching, and comes with a pump to capture single cells onto a chip. To remove air bubbles from the chip a vacuum chamber is required. If a vacuum chamber is unavailable, it is possible to order chips that are pretreated with a special coating to avoid bubble formation, and for which it is not necessary to remove bubbles in a vacuum chamber.

## Step-by-step method details

This section lists the major steps and provides step-by-step details and timing for each major step. Please use continuous number for this section.

### Trypsinize, count, and load cells

**Timing: 15 min**

This step describes how to prepare a suspension containing living single cells for the punch procedure. Depending on cell line and cell counting method used, this step may require more time. We count the hTERT RPE-1 cells with an automated cell counter such as the Bio-Rad TC20.

1. Filter serum-free medium using a 0.2 μm filter.
2. Trypsinize cells according to routine procedures.
3. Spin down cells to collect cells in a pellet (3 min, 400 rcf for hTERT RPE-1 cells).
4. Remove medium and reconstitute cell pellet in filtered serum-free medium.

***Optional:*** if serum was used to inactivate the trypsin, we recommend an extra washing step to remove traces of serum. This can be done by spinning down the cells a second time and reconstituting them again in fresh serum-free medium.

5. Count the cells.
6. Dilute ∼7500 cells in 200 μL of filtered serum-free medium.
7. If not all the cells harbor a fluorescent marker which can be used for automated selection, add Hoechst33342 (1:20.000 dilution) or another DNA dye to the cell suspension. This DNA staining is highly suitable for automated cell selection after the imaging step.

**CRITICAL:** When counting the concentration of cells, include also dead cells in the calculation. Dead cells will also block the pores! The chip contains only 6400 pores and adding a large excess of cells will inevitably result in multiple cells settling into one well. A modest excess of 1000–1500 cells is recommended however, because there is always a percentage of cells that don’t get captured, for example because they stick to the walls of the collection reservoir.

**CRITICAL:** Reconstitute the cell pellet in serum-free medium. Serum contains high amounts of proteins. These will stick to the needle, causing highly inefficient punching (see troubleshooting 1).

### Cell capture

**Timing: 15 min**

This step describes the capturing of single cells in the chip and mounting of the chip on the microscope.

8. Pipet the suspension containing the cells on the chip and apply a negative pressure of −20 mbar on chips with 5 μm pores, or −100 mbar on chips with 2 μm pores.
9. As soon as the chip runs almost dry (i.e., only a < 1 mm thick film of liquid on top of the chip), immediately stop the pump and clean the bottom of the chip by pipetting 4–5 times 1 mL of sterile, filtered PBS on the backside of the chip.
10. After the last wash, remove the last droplets of PBS by holding some absorbing paper to the corner of the bottom of the chip (Figure 2F). Avoid touching the chip.
11. Mount the chip on the Puncher and immediately pipet 150–200 μL of serum-free medium on the chip to keep the cells in a wet environment (Figure 2G).

**CRITICAL:** Carefully clean the backside of the chip, which faces the objective of the microscope. It should be completely dry and free of salt crystals or large dust particles. Contamination will result in poor quality of the reflection images. These images must be of high quality as they are essential for alignment and focusing of the chip, and evaluation of punch success.

### Image and select cells

**Timing: 30 min**

This step describes the imaging and selection of captured cells.

12. Align the chip according to the instructions in the puncher software.
13. Focus the chip using one of the fluorescence filters or the reflection channel.
14. Scan the relevant fluorescent channels, as well as the reflection channel. The reflection channel will be used to evaluate punching success. Fluorescent reporters typically require exposure times of 20–100 milliseconds. A full microwell chip scan using a 20× objective typically generates 225 images per channel and takes 15–20 min when scanning 4 different channels. When using a 40× objective, scanning time can be reduced by imaging only a part of the chip.
15. Select cells of interest via automated selection. Automated selection can be performed by setting intensity ranges in up to 3 channels simultaneously.

***Optional:*** Instead of selection based on microscopy images, a file with predefined cup numbers can be loaded to select cells of interest.

***Optional:*** Alternative to automated selection, cells can be manually selected. However, to minimize time between preparation of cell suspension and collection of cells in multi-well plates, automated selection is preferred. Images of punched cells are stored and can be linked to transcriptomic data in downstream analysis.

**Figure 3:**
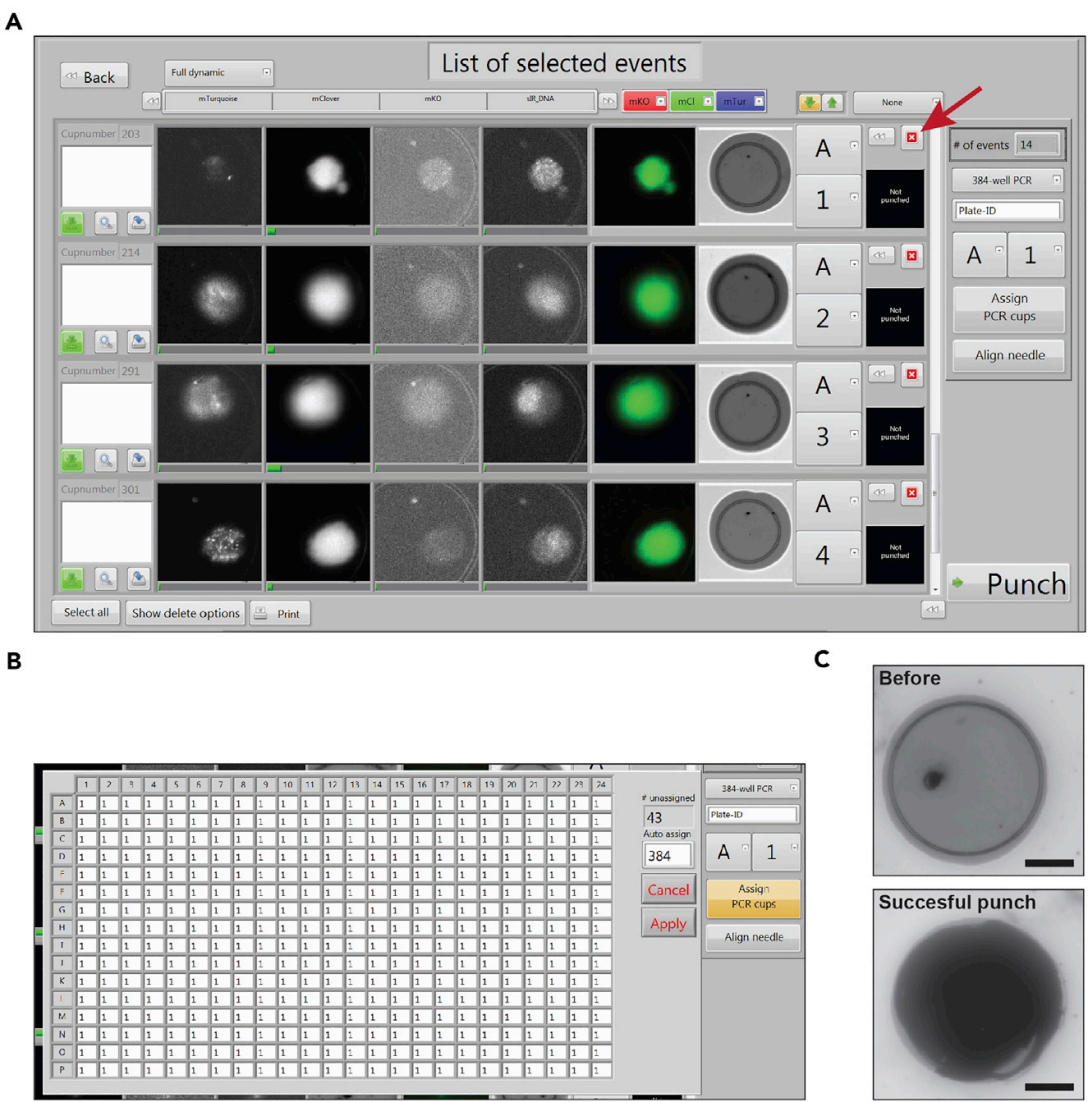
Cell selection and inspection of punch success (A) Screenshot from the VYCAP punch software showing a list of automatically detected cells. The list can be manually inspected and unwanted cells can be manually deleted (click button indicated by dark red arrow). (B) Screenshot from the VYCAP software showing the well assignment window. In this case the first 384 cells of interest were each assigned automatically to individual well positions. (C) Reflection images before and after a successful punch. Scalebar indicates 20 μm.

16. Scroll through the list of selected cells to visually inspect their quality (Figure 3A). Doublets or dead cells can simply be removed from the list.

### Punch cells into microwell plate

**Timing: 30 min**

This step describes how the cells are punched into 384-well plates compatible with an automated platform for CEL-Seq2 library preparation (Muraro et al, 2016). Wells of the collection plates contain primers and nucleotides in a volume of 100 nL, and 5 μL of mineral oil to avoid evaporation. Any desired alternative library preparation chemistry can in principle be used.

17. Take a ready-to-use 384-well plate containing CEL-Seq2 primers from the −20°C freezer, and briefly spin down the plate (1 min, 2000 rfc).
18. Remove the foil and place the plate on the puncher.

***Optional:*** a cooling block can be used to keep the 384-well plate cold to slow down potential RNA degradation.

19. Assign each selected cell to a specific well ID of the 384-well plate (A1 through P24) using the VYCAP software (Figure 3B).
20. Punch the cells.
21. After the last punch, immediately take the plate from the puncher, and seal it with adhesive foil.
22. Spin down the plate (1 min, 2000 rfc).
23. Immediately transfer the plate to a −80°C freezer.
24. Store at −80 degrees Celsius until library preparation.
25. Inspect the after images to confirm successful punching throughout the plate (Figure 3C). The number of correct punches should be ∼90% or higher; if not, please refer to troubleshooting.

**CRITICAL:** Taking after-images takes a substantial amount of time: 30 minutes for a full 384-well plate. Hence it is important to spin down and freeze the plate immediately after the last punch, and not wait until the after-images are all taken.

***Optional:*** Initially assign and punch a limited number of cells (5–10) and check the after-images to ensure successful punching. After this small test the entire plate can be punched. This allows the user to troubleshoot by quickly replacing or re-calibrate the needle if necessary.

## Expected outcomes

hTERT RPE-1 cells captured using this protocol, and further processed for single cell transcriptomics according to the CEL-Seq2 protocol (Muraro et al, 2016) typically yield libraries of 30.000 to 100.000 unique UMI counts, mapping to 4000–8000 different genes. This library complexity and sequencing depth is comparable to FACS sorted hTERT RPE-1 cells. We usually see that 100–150 of the wells from a fully punched 384-well plate contain sufficiently complex libraries for downstream RNA-seq analysis. This success rate is somewhat lower than FACS-sorted hTERT RPE-1 cells, which typically yields ∼200–250 high-quality libraries per 384-well plate.

## Quantification and statistical analysis

Quantitative image analysis can be done with ImageJ or any other imaging analysis software. An example of a macro that recursively quantifies mAG-hGeminin(1–110) in nuclear regions detected with the Hoechst33342 signal is included in this paper (Supplementary Macro). ROIs are drawn around the nuclei in the DAPI channel. The ROIs are then projected on the FITC channel to measure the mean fluorescence intensity within each nucleus. This information on fluorescent intensities can now be coupled into a metadata table using for example basic R coding in Rstudio, and then integrated with the RNA count data using the R-package Seurat (Satija et al, 2015).

## Limitations

In principle, any type of cell can be captured for single cell RNA-sequencing using this procedure. However, the cells should have a diameter within the range of 2–70 μm. The pores in the chip suitable for small cells have a diameter of 2 μm, and very small cells can thus slip through and disappear. The diameter of the cups is 70 μm, meaning that very large cells such as adult cardiomyocytes may not fit in the cups.

Another limitation is that the cells must not be suspended in a protein- or polysaccharide-rich environment, as these molecules tend to contaminate the needle and make the punch process highly inefficient. For example, we unsuccessfully tried to punch primary cells isolated from synovial fluid, which is highly rich in hyaluronic acid, even after extensive washing by multiple rounds of centrifugation and resuspension in PBS or basic medium.

A final limitation is that cells are captured alive, and the transcriptomes of the captured cells can change between the moment of isolation and punching. We are currently exploring the possibility to capture fixed cells. However, it remains to be seen if the effect of fixation on mRNA quality for single cell sequencing will be problematic. Besides ruling out the effect of processing time, an additional advantage would be that the morphology of the cells may be better preserved with a mild fixation. This might allow imaging of more complex morphologic cell structures.

## Troubleshooting

The main problems we encountered are related to a low percentage of successfully punched cups. If this percentage is well below the expected 90%, inspect the after-images to analyze which of the problems described below is the most likely underlying cause.

### Problem 1

**Figure 4:**
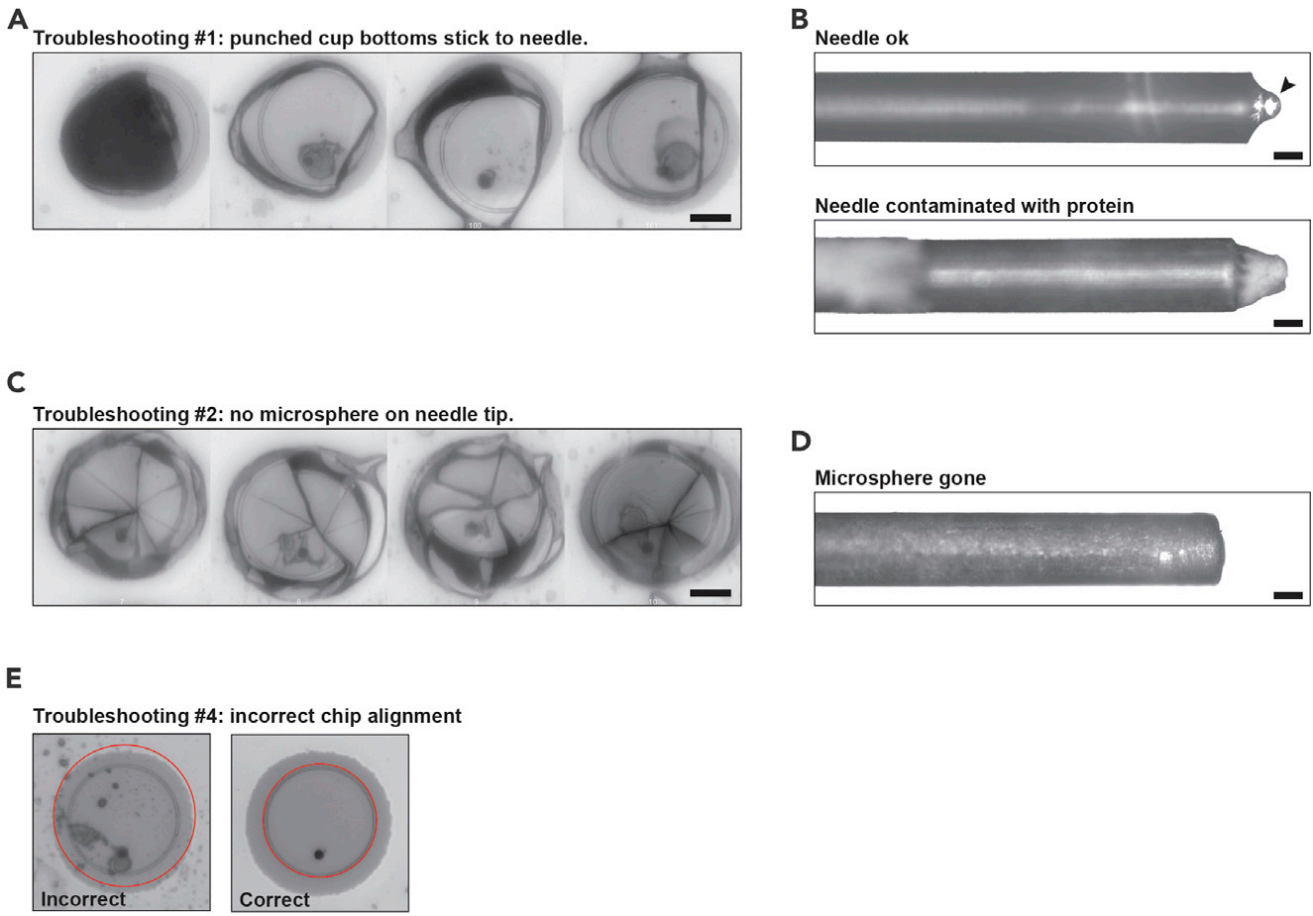
Troubleshooting (A) Filmstrip with reflection images from a failed experiment with a sticky needle. The bottoms do not fall, and stick to the chip when the needle returns to the start position. Scalebar: 20 μm. (B) Brightfield images of an intact clean punch needle (upper panel) and an example of a needle tip heavily contaminated with protein and hyaluronan (lower panel). Arrowhead: glass microsphere that is glued to the tip of the metal. Scalebar: 10 μm. (C) Filmstrip with reflection images from a failed experiment with a sharp punch needle; the wells get shattered, because the smooth glass microsphere has fallen off. Scalebar: 20 μm. (D) Brightfield image of a needle lacking the microsphere after accidentally hitting the metal of the chip between the wells. Scalebar: 10 μm. (E) Incorrect detection of the breaking edge of one of the corner cups during chip alignment. This is usually seen if the back-side of the chip is not properly cleaned (left panel), or if the contrast in the image is not high enough. The right panel shows correct detection.

After punching many well-bottoms stick to the chip, as can be seen in the after-images (Figure 4A).

This problem is most likely caused by contamination of either the needle or the chip with proteins (Figure 4B, lower panel). As a result, the tip of the punch needle becomes sticky. The needle breaks the bottom of the well, but the latter does not fall, and instead sticks to the chip as soon as the needle returns to the start position.

### Potential solution

Avoid high amounts of protein in the single cell suspension, for example by washing the cells multiple times with PBS after trypsinization. Resuspend the cells in serum-free medium. Finally, make sure to carefully wash the back side of the chip at least 5 times with 1 mL PBS before placing it on the microscope. The surface of the back side should look dry and clean after visual inspection. Additionally, after mounting the chip on the microscope the reflection channel can be used to further inspect the surface.

See step-by-step method details (steps 4–6 and 25).

### Problem 2

Many well-bottoms are shattered instead of punched, or not punched out at all (Figure 4D). This problem may occur when the round tip of the needle becomes sharp. We have for example seen this when the microsphere that is glued to the tip of the needle falls off. The tip is typically robust enough to punch a full 384-well plate. However, if the calibration was not done properly and the metal between the wells is hit during an unsuccessful punch, the microsphere will fall off (Figure 4G). The needle tip is now sharp metal, and it will not punch out the well correctly.

### Potential solution

Cancel the punch process and immediately replace the needle when it accidentally punches the metal of the chip. This can be done while the experiment is still ongoing. To prevent mis-punching, regularly recalibrate the needle after a limited number of punches. The latter can be done by checking the option to recalibrate after any desired number of punches in the start settings.

See before you begin (steps 11–15).

### Problem 3

The cell suspension liquid stops flowing through the chip after a few minutes when the vacuum is applied. This typically occurs if a large excess of cells is loaded on the chip.

### Potential solution

We advise to load a small excess of cells, but when the far majority of the cups is filled, there are insufficient pores to pull the remaining liquid through. Carefully remove the remaining cell suspension from the top of the chip and continue at step 9 of the step-by-step method details section.

See step-by-step method details (steps 5-6 and 8-9).

### Problem 4

During chip alignment the VYCAP software cannot detect the breaking edge of the wells at the corners of the chip (Figure 4E, left panel). This will result in incorrect alignment of the chip relative to the punch needle.

### Potential solution

This problem is typically seen if the back-side of the chip is not sufficiently not cleaned. The solution is to repeat steps 9 and 10 of the step-by-step protocol; in addition, try to carefully wipe the chip clean with a piece of lens-cleaning paper. The breaking edge can also be made more clearly visible by changing the exposure time, or by using the brightfield channel instead of the reflection channel for chip alignment.

See step-by-step method details (steps 9-10, and 12).

### Problem 5

Low percentage of cells with high-quality RNA-seq data, and many cells with a high percentage of mitochondrial gene counts (>30%). This is typically due to delays in the overall time between trypsinization and freezing down the plate with punched cells. The instability of mRNA makes it critical to work as fast as possible at all times.

### Potential solution

Reduce scanning time by 1) scanning only a part of the chip and 2) reducing exposure time during imaging as much as possible. The visual inspection of the list of automatically selected cups to remove unwanted ones -such as cups containing doublets- can be omitted during the experiments, and done retrospectively. Although these cups will be punched, they can thus be excluded from the final data analysis.

See step-by-step method details (steps 14 and 16) and expected outcomes.

## Resource availability

### Lead contact

Further information and requests for resources and reagents should be directed to and will be fulfilled by the Lead Contact, Bart Westendorp (b.westendorp@uu.nl).

### Materials availability

All materials newly generated in this study are available from the Lead Contact with appropriate regulatory clearances and a completed Materials Transfer Agreement.

### Data and code availability

Raw and processed single cell RNA-sequencing data generated in this study are available on Gene Expression Omnibus under accession number GSE146759.
